# Chronic exposure to polycyclic aromatic hydrocarbons alters skin virome composition and virus–host interactions

**DOI:** 10.1093/ismejo/wrae218

**Published:** 2024-10-25

**Authors:** Shicong Du, Xinzhao Tong, Marcus H Y Leung, Richard J Betts, Anthony C Woo, Philippe Bastien, Namita Misra, Luc Aguilar, Cécile Clavaud, Patrick K H Lee

**Affiliations:** School of Energy and Environment, City University of Hong Kong, Tat Chee Avenue, Kowloon, Hong Kong SAR, China; School of Energy and Environment, City University of Hong Kong, Tat Chee Avenue, Kowloon, Hong Kong SAR, China; Department of Biological Sciences, School of Science, Xi'an Jiaotong-Liverpool University, Ren’ai Road, Suzhou, 215123, P. R. China; School of Energy and Environment, City University of Hong Kong, Tat Chee Avenue, Kowloon, Hong Kong SAR, China; L’Oréal Research and Innovation, Raffles Quay, North Tower, 048583, Singapore; L’Oréal Research and Innovation, Rue Blaise-Pascal, Aulnay-Sous-Bois, 93600, France; L’Oréal Research and Innovation, Rue Blaise-Pascal, Aulnay-Sous-Bois, 93600, France; L’Oréal Research and Innovation, Rue Blaise-Pascal, Aulnay-Sous-Bois, 93600, France; L’Oréal Research and Innovation, Rue Blaise-Pascal, Aulnay-Sous-Bois, 93600, France; L’Oréal Research and Innovation, Rue Blaise-Pascal, Aulnay-Sous-Bois, 93600, France; School of Energy and Environment and State Key Laboratory of Marine Pollution, City University of Hong Kong, Tat Chee Avenue, Hong Kong SAR, China; Low-Carbon and Climate Impact Research Centre, City University of Hong Kong, Tat Chee Avenue, Hong Kong SAR, China

**Keywords:** skin, viromes, virus–host interactions, polycyclic aromatic hydrocarbons, auxiliary metabolic genes, viral lifestyle

## Abstract

Exposure to polycyclic aromatic hydrocarbons (PAHs) in polluted air influences the composition of the skin microbiome, which in turn is associated with altered skin phenotypes. However, the interactions between PAH exposure and viromes are unclear. This study aims to elucidate how PAH exposure affects the composition and function of skin viruses, their role in shaping the metabolism of bacterial hosts, and the subsequent effects on skin phenotype. We analyzed metagenomes from cheek skin swabs collected from 124 Chinese women in our previous study and found that the viruses associated with the two microbiome cutotypes had distinct diversities, compositions, functions, and lifestyles following PAH exposure. Moreover, exposure to high concentrations of PAHs substantially increased interactions between viruses and certain biodegrading bacteria. Under high-PAH exposure, the viruses were enriched in xenobiotic degradation functions, and there was evidence suggesting that the insertion of bacteriophage-encoded auxiliary metabolic genes into hosts aids biodegradation. Under low-PAH exposure conditions, the interactions followed the “Piggyback-the-Winner” model, with *Cutibacterium acnes* being “winners,” whereas under high-PAH exposure, they followed the “Piggyback-the-Persistent” model, with biodegradation bacteria being “persistent.” These findings highlight the impact of air pollutants on skin bacteria and viruses, their interactions, and their modulation of skin health. Understanding these intricate relationships could provide insights for developing targeted strategies to maintain skin health in polluted environments, emphasizing the importance of mitigating pollutant exposure and harnessing the potential of viruses to help counteract the adverse effects.

## Introduction

The skin is a physical barrier between the body and the environment and is inhabited by a microbial consortium consisting of bacteria [1, 2], fungi [1–3], and viruses, especially bacteriophages [2, 4, 5]. This consortium plays an indispensable role in modulating immune processes that are essential for maintaining skin health [6], and its composition varies depending on external environmental factors (e.g. season and pollution) and host physiological factors (e.g. age and genetics) [6–8].

Air pollution appears to play a significant role in various skin diseases, particularly in people living in urban environments [9]. For example, polycyclic aromatic hydrocarbons (PAHs) are ubiquitous air pollutants that substantially affect the skin [10]. PAH exposure has been implicated in the development of inflammatory skin diseases and cutaneous malignancies [11, 12], disorders in lipid metabolism in the stratum corneum and in skin protein composition [12], and skin cancer [13]. PAH exposure can also alter the diversity, taxonomic composition, and metabolic profiles of skin microbiome [14, 15], consequently affecting skin phenotype [15]. For example, high levels of PAH exposure have been linked to skin dryness and hyperpigmentation [15] and to a skin microbiome enriched in bacterial species capable of degrading xenobiotics [16]. Recent studies on skin metagenomes have revealed the composition and potential functions of the skin microbiome [2, 8, 15, 17] and the microbial taxonomy, functionality, and antimicrobial resistance profiles of skin-identified microbial sub-community clusters, denoted “cutotypes.” [17] Cutotype studies have clarified specific ecosystemic interactions between microbes, their functions, and their human hosts in the context of urbanization [15, 17].

The interactions between microbes and the skin’s immune systems are diverse and range from mutualism to pathogenicity [18]. Given that bacteriophages can modify the abundance and metabolism of their hosts, it is crucial to determine how bacteriophage–host interactions can impact healthy and inflammatory skin conditions. For example, *Corynebacterium* and *Staphylococcus* bacteriophages have been identified as key organisms affecting virus–host dynamics in healthy skin [4]. In the hair follicles of patients with acne, there is a reduced abundance of *Cutibacterium acnes* bacteriophages and their hosts compared to healthy subjects, and a positive correlation was found between the abundance of *C. acnes* bacteriophages and the severity of acne, which suggests that *C. acnes* bacteriophages play a significant role in maintaining skin health [19]. A recent study also revealed distinct phageomes in the skin of individuals with atopic dermatitis compared to those with healthy skin, suggesting a potential causal relationship between changes in viral and bacterial communities and the pathology of various skin conditions [20]. When exposed to environmental stresses, bacteriophages can alter their lifestyles and increase their replication and survival by adjusting their strategies for infecting bacteria, leading to increased spread of bacteriophage progeny [21, 22]. However, little is known about the diversity and composition of viruses associated with air pollution exposure levels, along with the mechanisms by which these viruses influence the abundance and metabolism of their bacterial hosts on the skin under different PAH levels.

**Figure 1:**
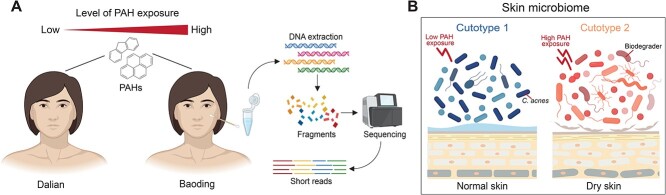
Differentiation of skin microbiomes into two cutotypes after exposure to low and high levels of polycyclic aromatic hydrocarbons (PAHs). (A) Workflow of sample processing. The illustration was made by BioRender. (B) The skin phenotypes associated with cutotypes 1 and 2 after exposure to low and high levels of PAHs. The illustration was drawn based on the results from the reference [15].

The skin microbiomes from the cheeks of 124 Chinese women residing in two cities in China, with various PAH exposure levels, differentiated into two distinct cutotypes [15]. Specifically, higher PAH exposure was associated with a loss of *C. acnes* and an enrichment of specific taxa, which in turn was associated with altered skin phenotypes, such as dry skin (Fig. 1). In this study, we analyzed viruses from the same 124 metagenomes [15] and hypothesized that the viruses associated with these two cutotypes differ in terms of diversity, composition, function, and lifestyle, that PAH exposure affects the ecological dynamics of the interactions between the viruses and their hosts, and that viruses can assist skin bacterial metabolism that was associated with skin phenotype. We speculated that there would be a strong coupling between the viruses and their bacterial hosts, and that viruses would assist skin microbial metabolism through progeny spread and gene insertion under high-PAH exposure, ultimately influencing the skin phenotype.

## Materials and methods

### Characteristics of participants and processing of metagenomic sequences

This research was approved by the Sino-German Cosmetics Institute Ethics Review Board (Protocol 2015–033-DY-024) and conducted in accordance with the principles of the Declaration of Helsinki developed by the World Medical Association. The metagenomic datasets [15] used in this study comprised 124 cheek skin samples collected from a cohort of female participants aged 25–45 who had resided in Baoding (more polluted; *n* = 60) or Dalian (less polluted; *n* = 64) for at least 15 years. These participants have been involved in a series of studies that have examined differences between them by city and in terms of the concentrations of PAHs in hair [23], the association of PAH exposure with facial aging signs [24], the taxonomic compositions of skin bacteria and fungi based on 16S rRNA gene and internal transcribed spacer region amplicon sequencing [14], metabolomic and proteomic profiles [25], and skin bacterial cutotypes based on metagenomic sequencing [15]. The sample collection and measurement of each participants’ skin clinical parameters (i.e. acne onset, age group, sebum concentrations, and facial pigmentation frequency) have been detailed in previous studies [14, 15, 23–25].

The cheek swabs and four negative controls (i.e. new sterile swabs) were processed and sequenced, as previously described [14, 25]. Briefly, a PowerSoil DNA Isolation Kit (MO BIO Laboratories, CA, USA) was used according to the manufacturer’s instructions [14] to extract genomic DNA from swabs that had been used to sample a cheek surface. Next, libraries were generated using the TruSeq DNA Sample Preparation kit v2 (Illumina Inc., San Diego, CA, USA) following the manufacturer’s protocols. The quality and yield of all libraries were verified prior to sequencing. Sequencing was then performed on a NovaSeq platform (Illumina Inc., San Diego, CA, USA) by SeqMatic (Fremont, CA, USA) to generate 150-bp paired-end reads. Quality control and assembly of reads into contigs were performed as previously described [14, 15]. Briefly, adapters were removed from the raw sequences using AdapterRemoval (v.2.3.1) [26]. Quality filtering and trimming were performed using KneadData (v.0.7.4) with the default parameters, and human sequences were removed using the human genome hg38 as the reference.

To remove contaminant sequences, any reads in a sample that could be mapped to contigs in the negative controls were removed using an in-house script, and any unpaired reads were further removed from the paired-end FastQ files using fastq-pair (v.1.0) [27]. Subsequently, taxonomic classification of the reads was performed using Kraken2 (v.2.0.7-beta; *k*-mer length = 35, confidence score threshold = 0) [28], species-level abundance estimation was performed using Bracken (v.2.6.1; threshold for filter = 0) [29], and all contaminating species identified by the R package “decontam” (v.1.12) based on prevalence mode with 0.1 as the significance threshold were then removed, as previously described [15]. After quality control, ~1.1 × 10^9^ paired-end clean reads were retained from all the samples. These reads were assembled into ~9.4 × 10^6^ contigs using the “assembly” function in MetaWRAP (v.1.2.1) [30].

### Recovery of viral bins and reconstruction of metagenome-assembled genomes

Viral contigs were detected using VirSorter2 [31] and DeepVirFinder [32], as previously described [33]. Variational autoencoders were employed for metagenomic binning: microbial species were binned using VAMB (v.3.1) [34], and bacteriophages were binned using PHAMB (v.1.0.1) [35]. VAMB was used to cluster contigs with a length > 1000 bp into putative microbial taxa with a depth matrix and to generate the tetranucleotide frequencies of the contigs, which were then parsed with PHAMB to generate putative viral bins. The quality of these bins was assessed using CheckV (v.0.8.1; database v.1.0) [36] with the “end_to_end” pipeline [37]. For the proviruses predicted by CheckV, we removed the host regions and retained only the proviral regions of the viral bins that were identified as proviruses for further analysis. The lifestyle of viral bins was predicted using BACPHLIP [38] and VIBRANT [39].

Reconstruction of metagenome-assembled genomes (MAGs) was performed as previously described [40]. Briefly, contigs with a length > 1000 bp in each sample were binned into MAGs using the “binning” function in MetaWRAP. The resulting MAGs were further refined using the “bin_refinement” function in MetaWRAP and then dereplicated into representative MAGs (rMAGs) using the “dereplicate” function with the default setting in dRep (v.3.2.2) [41]. The genomic quality of the rMAGs was assessed using the “lineage-wf” function in CheckM (v.1.1.2) [42]. Only medium-quality (completeness ≥50% and contamination ≤10%) and high-quality (completeness ≥75% and contamination ≤5%) rMAGs [43] were retained for downstream analysis. The taxonomies of rMAGs were assigned using the “gtdbtk classify_wf” function in GTDB-Tk (v.1.5.1) [44]. Different rMAGs that were assigned to the same species (95–99% average nucleotide identity [ANI]) were considered as separate strains. Phylogenetic analysis of rMAGs was performed using PhyloPhlAn3 [45] and visualized using iTOL (https://itol.embl.de).

### Viral bin clustering and taxonomic assignment of viral operational taxonomic units

All viral bins with a completeness ≥50% were clustered into species-level viral operational taxonomic units (vOTUs) on the basis of a 95% ANI in the >85% alignment fraction, relative to the shorter sequences, using centroid-based clustering [37]. Genus- and family-level vOTUs were generated using a combination of shared genes and amino acid identity (AAI) based on Markov clustering [46], as described previously [37]. Briefly, viral bins with <20% AAI or < 10% shared genes and an inflation factor of 1.2 were clustered into family-level vOTUs, whereas those with <50% AAI or < 20% shared genes and an inflation factor of 2.0 were clustered into genus-level vOTUs.

The open reading frames (ORFs) in the vOTUs were predicted using Prodigal (v.2.6.3) [47] with the parameter “-p meta.” The protein-coding sequences of vOTUs were taxonomically assigned using the majority-rule approach, as previously described [37]. Briefly, the taxonomy (based on the International Committee on Taxonomy of Viruses) of the top hit in the IMG/VR (v4) database [48] obtained using DIAMOND (v.0.9.32; options: –query-cover 50 –subject-cover 50 –E-value 1e^−5^ –max-target-seqs 1000 –top 25) was transferred to each protein. Each vOTU was assigned to the lowest taxonomic rank of >70% of the annotated proteins. For family- and genus-level taxonomy assignment, a vOTU must have at least two annotated proteins with >30% average AAI or three annotated proteins with >40% average AAI, respectively, aligned to a reference genome in the IMG/VR database [37].

### Estimation of coverage of rMAGs and vOTUs

The clean reads from all the metagenomes were mapped to the vOTUs using Bowtie2 (v.2.4.4) with the default parameters. The “filter” function in BamM (v.1.7.3) was used to remove low-quality mappings, and reads that were aligned over ≥90% of their length at ≥95% ANI were retained. The vOTUs with ≥70% of their length covered by the reads were selected using a Python script in Read2RefMapper (v.1.1.0), and the average per-base-pair coverage of each vOTU in each sample was generated using the “parse” function in BamM with the parameter “tpmean” to remove the highest and lowest 10%-coverage regions. To normalize the coverage of the vOTUs across all samples, the number of reads in each sample was divided by the average number of reads across all samples and multiplied by the coverage retained by BamM, as previously described [49].

To calculate the coverage of each rMAG in all samples, the clean reads from all metagenomes were mapped to each rMAG using the “make” function in BamM. Low-quality read mappings (<75% of the aligned length of each read and <95% ANI) were removed using the “filter” function in BamM. The coverage of each contig in each rMAG in a sample was calculated as the average number of reads aligned to each position in the contigs after removal of the highest and lowest 10%-coverage regions via the BamM “parse” function in the “tpmean” mode. Subsequently, the coverage of each rMAG in all samples was calculated as the average coverage of all its binned contigs, with each contig weighted by its length in base pairs. The coverage of rMAGs across all samples was normalized by read numbers following the method described above for the vOTUs.

### Functional annotation of vOTUs and rMAGs

The ORFs of vOTUs were functionally annotated against the KO database using KOfamscan (https://www.genome.jp/tools/kofamkoala/) [50] with an E-value <0.01. The functions of KOs associated with the arginine biosynthesis pathway and xenobiotic degradation were manually curated. The coverage of genes in vOTUs was calculated by multiplying the corresponding coverage of vOTUs with the sequence coverage of genes that were aligned to vOTUs, using a BLASTn alignment threshold of identity ≥70% and an E-value ≤10^−5^. Phage-encoded auxiliary metabolic genes (AMGs), lysogenic marker (integrase), and lytic markers ((endo)lysin, holin, and peptidoglycan hydrolase) were annotated using the viral mode of DRAM (v.1.4.0) [51] and VIBRANT (v.1.2.1) [39] with the default parameters. The potential functions of AMGs were further assessed based on their predicted protein structures. The secondary and tertiary structures of AMGs were predicted by conducting a homology search against template models in the Protein Data Bank [52] and the AlphaFold Protein Structure Database [53] using Phyre2 (v.2.0) [54] with an alignment coverage of >65%. The BPROM (http://www.softberry.com/) and FindTerm (http://www.softberry.com) servers were used to predict the promoters and terminators of AMGs, respectively.

The ORFs of rMAGs were predicted using Prokka (v.1.14.6) [55] and their functions were annotated using EggNOG-mapper (v.2.1.11) [56] with the “Diamond” option and an *E*-value ≤10^−3^. Virulence factor genes (VFGs) were determined by aligning all ORFs to the Virulence Factor DataBase [57] using BLASTp with an E-value ≤10^−5^, an AAI of ≥80%, and a query coverage of ≥80% [58].

### Determination of virus–host links

Three complementary *in silico* methods were adopted to improve the accuracy of host prediction, namely methods that find exact or close matches of CRISPR spacers and integrated viral fragments in host genomes and a method that finds consistent *k*-mer signatures, as described previously [33]. Briefly, CRISPR spacers in the rMAGs were identified using CRT (v.1.2) [59] and PILER-CR (v.1.0.6) [60]. The identified spacers were compared against the vOTUs using BLASTn with ≤1 mismatch, 100% identity, and 100% coverage to establish virus–host links. Genomic regions shared between vOTUs and rMAGs were detected using BLASTn with an identity ≥90%, a hit length ≥ 1000 bp, and an *E*-value ≤0.001. The *k*-mer frequencies of sequences from rMAGs and vOTUs were estimated using WIsH (v.1.1) [61], as previously described [33]. The host predicted by WIsH that had the lowest *P* value (≤ 10^−5^) was retained for each vOTU. All the hosts predicted by the above-mentioned three methods were retained for use in analyzing virus–host interactions. The network of virus–host links was visualized using Cytoscape (v.3.9.0) [62].

The virus-to-host abundance ratio (VHR) of a vOTU and its linked host based on its rMAG was calculated by dividing the respective coverages in a sample. The correlations between the relative abundances of vOTUs and their predicted hosts, and between PAH concentrations and VHRs, were estimated by Pearson’s correlation analysis.

### Detection of CRISPR/Cas and anti-CRISPR/Cas systems

The CRISPR/Cas system in a rMAG was identified using CrisprCasTyper (v.1.2.3) [63]. The counter-defense systems in a vOTU, including anti-CRISPR (Acr) homologs, anti-CRISPR-associated (aca) genes, and Aca-like proteins, were identified using AcaFinder (version: Oct 15, 2022) [64]. To examine the phylogeny of Acr homologs, all the predicted Acr homologs were compared with the core dataset in anti-CRISPRdb (v.2.2) [65] using BLASTp with an identity ≥60% and an E-value ≤10^−5^. Multiple sequence alignments between the predicted Acr homologs and the reference Acr proteins obtained from the core dataset were generated using FAMSA (v.1.5.12) [66] and trimmed using trimal (v.1.4) [67] to retain positions with <50% gaps. A maximum-likelihood phylogenetic tree was constructed with the aligned sequences using FastTreeMP (v.2.1.11) with the auto model and then visualized using iTOL.

### Measurement of PAHs and other compounds in hair samples

The participants’ levels of exposure to pollutants were assessed by analyzing hair samples, as reported previously [23, 68]. Briefly, a hair was cut as close to the skin as possible from the occipital area of each participant and decontaminated, and then the first 12 cm (from the skin end) of the sample were analyzed. Specifically, this portion of the sample was pulverized, hydrolyzed, and extracted, and the extract was analyzed by gas or liquid chromatography–tandem mass spectrometry to determine its concentrations of 15 PAHs, nicotine, and cotinine [23].

### Measurement of skin metabolites

The metabolites in skin samples collected from participants were analyzed using untargeted metabolomic techniques, as reported previously [25]. Sample preparation involved adding recovery standards for quality control. Metabolites were extracted, and the resulting extracts were divided into five fractions for analysis via liquid chromatography-mass spectrometry. This analysis utilized high-resolution mass spectrometry, interfaced with an electrospray ionization source, and Orbitrap mass analyzer. Compounds were identified by comparing them to a reference library based on various characteristics, including retention time, molecular weight, preferred adducts, in-source fragments, and mass spectrometry spectra.

### Statistical analysis

All statistical analyses were conducted using R (v.4.2.2), and a *P* value of <0.05 was considered to indicate a statistically significant difference. Rarefaction was performed at a depth of 33 538 sequences, determined by the sample with the fewest sequences, using the “rrarefy” function in the R package “vegan” (v.2.6–4) [69]. α-diversity, based on this rarefaction depth, was assessed using the Shannon index, calculated with the “diversity” function in the same package. One-way analysis of variance (ANOVA) with Tukey’s honestly significant difference test was used to compare the differences between the α-diversities of cutotypes. Principal coordinate analysis based on Bray–Curtis dissimilarity was performed using the “vegdist” function in the R package “vegan,” and the multivariate homogeneity of variances was analyzed to test for differences in multivariate dispersions between cutotypes using the “betadisper” function in the same R package. The influences of factors (cutotype, city, acne onset, age group, sebum concentrations, and facial pigmentation frequency) on skin viral communities were assessed using a permutational multivariate analysis of variance (PERMANOVA) through the “adonis2” function in the R package “vegan.” Canonical correspondence analysis between PAH concentrations and viral taxonomic compositions was performed using the R package “vegan.” The Procrustes test was utilized to determine congruency between taxonomic and functional compositions using the “protest” function in the R package “vegan.” The associations of rMAGs, vOTUs, and functions (i.e. KOs and KEGG pathways) with cutotypes were determined via a generalized linear model through MaAsLin2 [70], with an adjusted *P* value (i.e. *q*-value) of <0.05 as the threshold for statistical significance. PLS-PM was performed using the R package “plspm” (v.0.5.0), and the Euclidean distance matrix of PAHs and the Bray–Curtis dissimilarity matrix of bacterial and viral compositions were used to account for the effects of PAHs on bacteria and viruses. The correlations between the specific KOs and their associated metabolites were assessed using Pearson’s correlation analysis. Two-tailed Wilcoxon rank sum test (WRST) with Benjamini–Hochberg (BH) adjustment was used to determine statistical differences between the two groups.

## Results

### High levels of PAH exposure altered skin virome compositions

**Figure 2:**
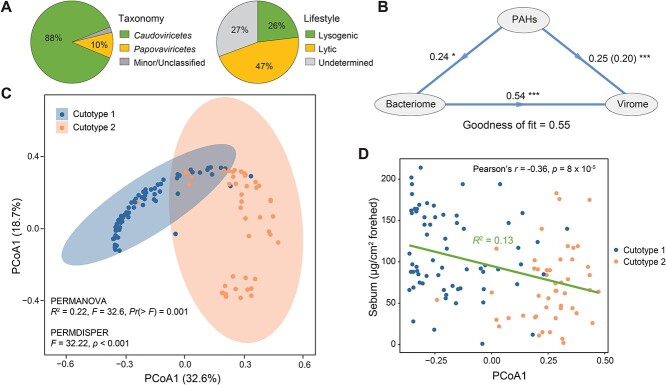
Overview of cheek skin virome. (A) Taxonomy and lifestyle of viruses. (B) Relationships between PAHs, bacteriome, and virome based on partial least squares path modeling. The numbers next to the lines and in the brackets denote statistically significant direct and indirect path coefficients, respectively (^*^P < .05 and ^*^^*^^*^P < .001). (C) Principal coordinate analysis of the Bray–Curtis dissimilarity matrix of vOTUs in all cheek skin samples. Cutotypes 1 and 2 were defined based on the microbes in the samples. Ellipses represent 95% confidence intervals. (D) Pearson’s correlation between forehead sebum concentration and the first dimension of the viral community compositional principal coordinate axis (i.e. PCoA1). The linear regression line and coefficient of determination (*R*^2^) are shown.

Examination of the 124 cheek skin samples enabled the recovery of 11 780 putative viral bins of >5 kb (Table S1). After quality filtering, 267 viral bins with >50% completeness and no contamination were obtained, comprising medium-quality (50%–90% completeness; *n* = 125), high-quality (> 90% completeness; *n* = 123), and complete (*n* = 19) bins (Table S1). Additionally, 30 complete, 57 medium-, and 49 high-quality single viral contigs were identified, comprising only about half of the number of viral bins identified (Fig. S1A). However, rarefaction analysis indicated that the 264 viral bins did not fully represent the skin viromes (Fig. S1B). Subsequently, clustering of the 267 viral bins revealed 238 species-level vOTUs, 88 of which were in at least two of the samples. The viral bins were also clustered into 134 and 89 genus- and family-level vOTUs, respectively. The rarefaction curves of the vOTUs were unsaturated at all taxonomic ranks, suggesting that additional samples would be required to capture skin virome diversity (Fig. S1C). Nevertheless, the number of vOTUs identified in our study is comparable to those reported in other skin studies [2, 20]. Most of the vOTUs (88%) were classified as belonging to the class *Caudoviricetes* (tailed bacteriophages), demonstrating that bacteriophages comprise a significant proportion of skin viruses [4, 5] (Fig. 2A). Moreover, most of the viruses were unclassifiable at lower levels, similar to the viromes that have been found at other body sites [2, 4]. Additionally, bacteriophages with a lytic lifestyle were predominant, comprising 47% of the virome (Fig. 2A).

Previously, metagenomic analysis of the 124 samples revealed that the skin microbiomes differentiated into cutotypes 1 and 2, which differed substantially in terms of diversity, and taxonomic and functional compositions (Fig. 1) [15]. Cutotype 2 was associated with high levels of PAH exposure, featured a higher bacterial diversity and a lower relative abundance of *Cutibacterium acnes*, and was found in those with a dry and dull skin phenotype, whereas cutotype 1 was associated with low levels of PAH exposure, featured a lower bacterial diversity and a higher relative abundance of *C. acnes*, and was found in those with a normal skin phenotype [15] (Fig. S2A). The average concentration of the 10 high-molecular-weight PAHs (i.e. > 200 g/mol) was significantly greater in the hair of those with cutotype 2 (75.2 ± 28.4 pg/mg hair) than of those with cutotype 1 (55.9 ± 28.5 pg/mg hair) (WRST, two-sided, *p*_adj_ = 0.008), whereas there was no difference in the average concentration of the seven low-molecular-weight PAHs (i.e. < 200 g/mol) between the cutotypes (WRST, two-sided, *p*_adj_ = 0.48) (Fig. S2B). Canonical correspondence analysis showed that the concentrations of most PAHs were significantly associated with vOTUs (Table S2). Partial least squares path modeling of the relationships between the bacteria, viruses, and PAHs showed that PAHs were significantly and positively associated with both bacteria (0.24) and viruses (0.44) (direct (0.25) + indirect (0.20) path coefficients) (Fig. 2B). The indirect path coefficient between PAHs and viruses, mediated by bacteria, was 0.20 (direct path coefficients between PAHs and bacteria (0.24) × those between bacteria and viruses (0.849)) (Fig. 2B). This is similar in magnitude to the direct path coefficient (0.25), indicating the crucial role played by bacteria in the relationship between PAHs and viruses.

Given that bacteria and viruses may interact when exposed to PAHs, the viruses associated with the two cutotypes were further explored. Principal coordinate analysis confirmed that the viruses could be separated into two distinct clusters that corresponded with the bacterial cutotypes (PERMANOVA, *R*^2^ = 0.22, *p* < 0.001; Fig. 2C). This was supported by the result of the permutation test for homogeneity of multivariate dispersions (*F* = 32.2, *p* < 0.001). The abundances of lysogenic and lytic lifestyles, respectively, were marginally different (WRST, two-sided, *p*_adj_ = 0.058) between the two cutotypes (Fig. S3A). The concentration of forehead sebum was negatively correlated with the first dimension of the viral community compositional principal coordinate axis (Pearson’s *r* = −0.36, *p* = 8 × 10^−5^) (Fig. 2D), suggesting skin phenotypes and viral community compositions were partially linked. The viruses associated with cutotype 1 had a significantly higher Shannon diversity (ANOVA, *p* < 0.001; Fig. S3B) than those associated with cutotype 2. Similar numbers of viral bins were identified from samples of cutotype 1 (*n* = 133) and cutotype 2 (*n* = 134), but a substantially greater number of vOTUs were enriched in cutotype 1 (*n* = 34) than in cutotype 2 (*n* = 3) (WRST, two-sided, *p*_adj_ < 0.05) (Fig. S3C). The genus *Pahexavirus* was marginally significantly higher in relative abundance in cutotype 1 than in cutotype 2 (WRST, two-sided, *p*_adj_ = 0.055; Fig. S3D), whereas the family *Zierdtviridae* was enriched in cutotype 2 (WRST, two-sided, *p*_adj_ = 0.038; Fig. S3D). The virus species evenness varied significantly between the two cutotypes (ANOVA, *p* < 0.05), but the average evenness of both cutotypes was >0.9 (Fig. S3B), suggesting that no specific viral populations were dominant.

### High levels of PAH exposure decreased potential virome functions

The potential functions of viruses were examined to determine how differences in taxonomic composition between cutotypes caused variations in their functional profiles. Similar to skin bacteria [15], the taxonomy and functions of the viral samples were congruent in cutotypes 1 and 2 (Pearson’s *r* = 0.991, *P* = .001; Fig. S4A), suggesting that samples with similar taxonomic compositions tended to have similar functions. Analysis of the vOTUs identified 18 842 ORFs, which were clustered into 14 269 *de novo* ORF clusters. Their accumulation curves were unsaturated, indicating that the viromes contained highly diverse functions (Fig. S4B). The ORF clusters with >10 genes mostly encoded proteins with DNA replication, repair, and recombination functions (Fig. S4C). Compared with factors such as skin condition or geographic location, cutotype better accounted for functional differences between viromes (Table S3).

**Figure 3:**
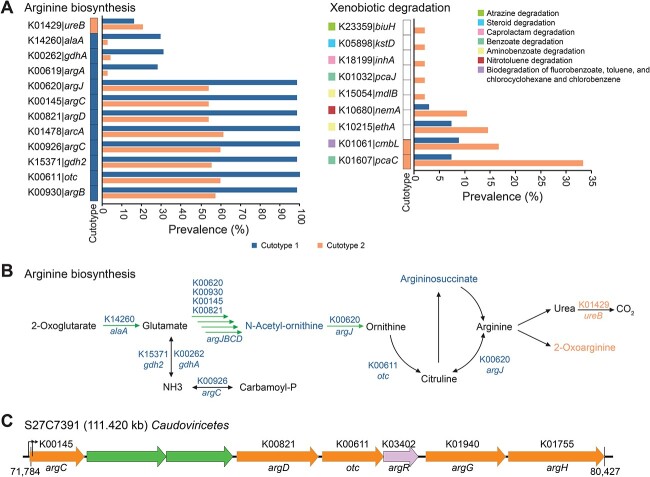
Functional profile of skin viromes. (A) The prevalence of enriched KEGG Orthologies (KOs) involved in arginine biosynthesis (left panel) and xenobiotic degradation (right panel) pathways between the two cutotypes, as analyzed using MaAsLin2. The vertical bars indicate the enrichment of these KOs in cutotype 1 and cutotype 2. (B) KOs and their related metabolites involved in the arginine biosynthesis pathway are shown. KOs highlighted in color are those that are enriched in their respective cutotypes. Similarly, metabolites shown in color indicate significant positive correlations with the enriched KOs in their respective cutotypes. (C) Schematic illustration of genes in the arginine biosynthesis pathway carried by a *Caudoviricetes* virus in cutotype 1. Different colors represent various functional modules, and arrows indicate predicted terminators.

Multivariate analysis of the Kyoto Encyclopedia of Genes and Genomes (KEGG) Orthology (KO) gene families of viruses in cutotypes 1 and 2 revealed differential enrichment of 755 KOs (675 in cutotype 1 and 80 in cutotype 2; Table S4). The KOs enriched in cutotype 1 were involved in a diverse range of metabolic functions related to biosynthesis, whereas cutotype 2 was characterized by a more restricted set of metabolic functions (Table S4). Previous research indicated that the arginine pathway is enriched in the skin microbiome of cutotype 1 [15]. Consistent with this, our findings revealed differential enrichment of 12 KOs involved in the arginine biosynthesis pathway between cutotypes 1 and 2 of the skin viromes, with 11 KOs significantly enriched in cutotype 1 (WRST, two-sided, *p*_adj_ < 0.05; Fig. 3A and Table S4). Significant positive correlations between these 11 KOs and two metabolites associated with arginine metabolism were observed in cutotype 1 (Fig. 3B). Specifically, N-delta-acetylornithine showed significant correlations with all 11 KOs (Pearson’s *r* = 0.19–0.44, *P* < .05) and argininosuccinate was significantly correlated with K14260 [*alaA*] (Pearson’s *r* = 0.19, *P* < .05) (Table S4). In contrast, the single KO related to arginine metabolism (K01429 [*ureB*]) that was enriched in cutotype 2 was significantly correlated with 2-oxoarginine (Pearson’s *r* = 0.19, *p* < 0.05) (Fig. 3B). Our analysis also revealed that a vOTU in cutotype 1 can carry a relatively complete arginine biosynthesis pathway. Specifically, one *Caudoviricetes* virus (S27C7391) from cutotype 1 contains five KOs (i.e. K00145 [*argC*], K00821 [*argD*], K00611 [*otc*], K01940 [*argG*], and K01755 [*argH*]) that encode proteins involved in this pathway (Fig. 3C). Additionally, the repressor (i.e. *argR*) of the *arg* operon [71] and the predicted promoter responsible for regulating transcription were identified in S27C7391 (Fig. 3C), suggesting a functional arginine biosynthesis operon.

As high-level PAH exposure enriches xenobiotic degradation functions in skin bacteria of cutotype 2 [15], we investigated whether a similar effect might also occur in the viromes. Nine KOs associated with xenobiotic degradation were identified in the viromes, all of which were more prevalent in cutotype 2 compared to cutotype 1, with five of these KOs completely absent in cutotype 1 (Fig. 3A). Among the remaining four KOs, K01607 [*pcaC*]), associated with benzoate degradation, and K01061 [*cmbL*], related to biodegradation of fluorobenzoate, toluene, and chlorocyclohexane and chlorobenzene, were enriched in the viruses found in cutotype 2. Benzoate is a key intermediate in the biodegradation of many low- and high-molecular-weight PAHs [72], and the metabolites associated with benzoate detected in the skin samples included hippurate, 4-hydroxybenzoate, methyl- and propyl-4-hydroxybenzoate, and p-Cresol sulfate. Consistently, KO (K01607 [*pcaC*]) showed a significant positive correlation with hippurate (Pearson’s *r* = 0.19, *P* = .0041). These findings suggest that the viruses in cutotype 2 may confer adaptive advantages for xenobiotic biodegradation on their bacterial hosts when exposed to high concentrations of PAHs.

### Numbers of virus–host interactions varied with PAH exposure

To elucidate the responses of virus–host interactions under PAH exposure, the putative links between viruses and their bacterial hosts in all the cheek skin samples were examined. The *in situ* bacterial hosts were represented by rMAGs reconstructed from the samples, i.e. 77 medium-quality and 35 high-quality rMAGs (Fig. S5A and Table S5). The predominant rMAGs were affiliated with the phyla *Actinomycetota* (*n* = 58) and *Proteobacteria* (*n* = 46). Only 10 rMAGs were enriched in cutotype 1, with *C. acnes* (*n* = 7) predominating, whereas 95 were enriched in cutotype 2, with *Micrococcus luteus* (*n* = 9) predominating (Fig. S5A and Table S5). This result is consistent with the higher bacterial diversity and lower *C. acnes* relative abundance observed in samples from cutotype 2 versus cutotype 1, based on metagenomic short reads [15]. Given that virulence within the skin microbiome can significantly impact the development and manifestation of various skin phenotypes [6], the global virulence in the rMAGs was examined. VFGs were found in 64 rMAGs, 62 of which belonged to those enriched in cutotype 2 and only two of which belonged to those enriched in cutotype 1 (each carrying only a single VFG) (Fig. S5B and Table S5).

Of the vOTUs analyzed, 88% (211/238) were linked to 91% (102/112) of the reconstructed rMAGs, resulting in 723 virus–host links (Fig. S5C and Table S6). After normalizing the number of links to the number of enriched rMAGs in each cutotype, significant differences were observed, with cutotype 1 having 30 links per rMAG and cutotype 2 having only 3 per rMAG. This discrepancy was mainly due to a limited number of rMAGs (*n* = 10) enriched in cutotype 1, all belonging to the genus *Cutibacterium*, whereas cutotype 2 had a significantly larger number of enriched rMAGs (*n* = 84) from diverse genera (Fig. S5C and S5D). Among these links, the only exact CRISPR spacer-match was between a temperate virus (S19C11122) and the human pathogen *Acidovorax temperans* (LOR022Cbin2) in a sample from cutotype 2, representing strong evidence of viral infection in cutotype 2. Nevertheless, this pathogen could be infected by another virus (S4C2191) with a lytic lifestyle (Table S6).

**Figure 4:**
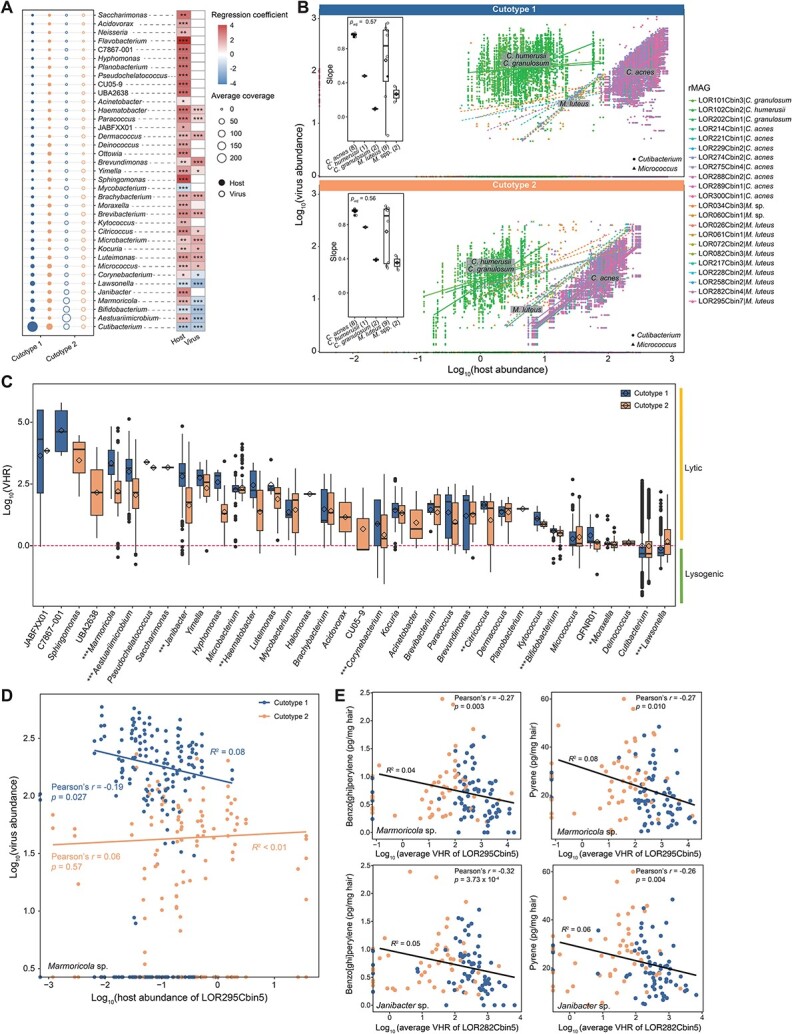
Numbers of virus–host interactions in the two cutotypes. (A) Left panel: Average relative abundances of viruses and their linked bacterial hosts. Right panel: Heatmap of the regression coefficient from the general linear model of MaAsLin2 showing differentially abundant hosts and viruses between the two cutotypes (Wilcoxon rank sum test (WRST), two-sided, ^*^p_adj_ < .05, ^*^^*^p_adj_ < .01, ^*^^*^^*^p_adj_ < .001). Only genera enriched or depleted in cutotypes 1 and 2 are shown. A positive coefficient indicates the degree of enrichment, whereas a negative coefficient indicates the degree of depletion in cutotype 2. (B) Pearson’s correlations of relative abundances (log_10_ scale) between viruses and their linked hosts of various *Cutibacterium* species (including eight *C. acnes* strains) and *Micrococcus* species (including nine *M. luteus* strains) in cutotypes 1 and 2. The inset boxplots show the slopes of linear regression for strains of each species. (C) Virus-to-host ratio (VHR) at the bacterial genus level for each virus–host pair. The statistical significance of the difference in VHRs between cutotypes 1 and 2 is indicated on the *x*-axis (WRST, two-sided, ^*^p_adj_ < .05, ^*^^*^p_adj_ < .01, ^*^^*^^*^p_adj_ < .001). A VHR > 1 indicates that the viruses tended to have a lytic lifestyle, whereas a VHR < 1 indicates that the viruses tended to have a lysogenic lifestyle. (D) Pearson’s correlations of the relative abundances (log_10_ scale) between viruses and their linked host (i.e. a *Marmoricola* sp.) in the two cutotypes. (E) Pearson’s correlations between the average VHR of selected *Marmoricola* and *Janibacter* species and the measured concentrations of polyaromatic hydrocarbons (PAHs; only the VHR–PAH pairs with the two largest Pearson’s correlation coefficients (*r*) are shown). Linear regression lines and coefficients of determination (*R*^2^) are shown in panels d and e. In each box-and-whisker plot, the box indicates the median, first quartile, and third quartile; the whiskers span 1.5 times the interquartile range; and the diamond indicates the mean. Points that lie beyond the whiskers indicate outliers.

To clarify the ecological role of virus–host interactions in the two cutotypes, the lineage-specific correlations between viruses and their host abundances and the VHRs were analyzed. Several overabundant (WRST, two-sided, *p*_adj_ < 0.05) viruses in cutotype 1 or 2 were linked to hosts such as *Cutibacterium*, *Aestuariimicrobium*, *Bifidobacterium*, and *Marmoricola* with a high relative abundance and prevalence (Fig. 4A). Among these, *Cutibacterium*, particularly *C. acnes*, was the most susceptible to viral infection (Fig. S5D), showing a predominant average relative abundance in cutotype 1 (Fig. 4A). The relative abundances of the eight *C. acnes* strains and their linked viruses were highly similar in cutotype 1 and cutotype 2, whereas other *Cutibacterium* species exhibited significantly different relative abundances of viruses and hosts (WRST, two-sided, *p*_adj_ = 0.012) across both cutotypes (Fig. 4B). A similar pattern of relative abundances was observed for the nine *M. luteus* strains compared to other *Micrococcus* species, all of which were enriched in cutotype 2 (Fig. 4A and 4B).

**Figure 5:**
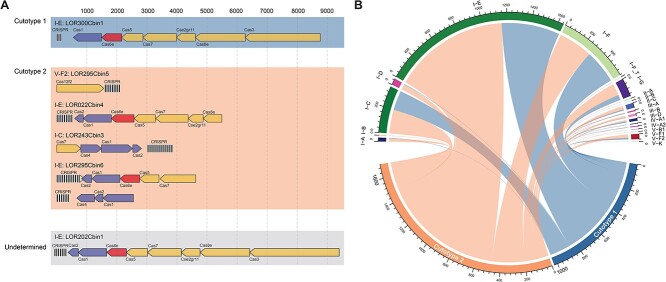
Clustered regularly interspaced short palindromic repeat (CRISPR)/CRISPR-associated protein (Cas) systems and predicted anti-CRISPR (Acr) proteins in the skin viromes. (A) The six representative metagenome-assembled genomes (rMAGs) in cutotypes 1 and 2 carried seven complete CRISPR/Cas systems, with one rMAG enriched in cutotype 1 and four rMAGs enriched in cutotype 2. (B) Circos diagram illustrating the type and number of Cas operons identified in the contigs of the two cutotypes.

In both cutotypes, the average VHRs for most genera were > 1, indicating that the viruses tended to utilize lytic infection to target and lyse a majority of the bacteria. However, the average VHRs for a few genera, including those belonging to *Cutibacterium* and *Micrococcus*, were close to 1 (Fig. 4C). Most of the genera in cutotype 2, including some with biodegradation ability (e.g. *Janibacter* [73] and *Marmoricola* [74]) had significantly lower VHRs than those in cutotype 1 (WRST, two-sided, *P*_adj_ < .05) (Fig. 4C), and the correlations between the relative abundances of viruses and their hosts sometimes differed between cutotypes. For example, the relative abundance of a *Marmoricola* species (LOR295Cbin5) was significantly and negatively correlated with its linked viruses in cutotype 1 (Pearson’s *r* = −0.19, *P* = .027), whereas it was positively correlated with its linked viruses in cutotype 2, albeit nonsignificantly (Pearson’s *r* = 0.06, *P* = .57) (Fig. 4D). To further support this observation, we compared the relative abundance of viruses linked to *Marmoricola* spp. and *Janibacter* spp. carrying lysogenic or lytic markers between the two cutotypes. The results showed that viruses linked to these genera predominantly carried a lysogenic marker and were primarily found in cutotype 2 (Fig. S6), which aligns with the lower VHRs observed in these genera. To further investigate the effects of PAHs on virus-host interactions, the concentrations of PAHs were correlated with the VHRs. Exposure to both low- and high-molecular-weight PAHs had differential effects on the virus-host interactions of 24 rMAGs (Fig. S7A). The concentrations of many of the high-molecular-weight PAHs (e.g. pyrene, benzo[*a*]anthracene and benzo[*ghi*]perylene) were significantly and negatively correlated with the average VHRs of 21 rMAGs (*P* < .05; Fig. S7A and S7B), especially those for a *Marmoricola* species (Fig. 4E), suggesting that PAH exposure and virus–host interactions are partially linked.

### Number of CRISPR-Acr interactions were increased under high-PAH exposure

The interactions between viruses and their hosts were further examined by investigating the relationship between the antibacteriophage immune system, specifically the CRISPR/Cas system that bacteria have evolved to protect themselves from bacteriophage infections, and the counter-defense mechanism, specifically the Acr proteins, employed by bacteriophages [75]. Seven complete CRISPR/Cas operons were identified in six rMAGs, five of which were in the rMAGs that were enriched in cutotype 2 (Fig. 5A). This is consistent with a greater number of Cas operons in the contigs of cutotype 2 (*n* = 1709) than in those of cutotype 1 (*n* = 1027) (Fig. 5B). The rMAGs carrying a Cas operon were primarily associated with *Actinomycetia* and *Alphaproteobacteria*, predominantly featuring the type IE Cas operon (Fig. S5A), which is consistent with the contig results (Fig. 5B). Additionally, the rMAGs enriched in cutotype 2 exhibited a wider array of Cas operons, suggesting that bacterial hosts in this cutotype have evolved more strategies to protect themselves from viral infections under high PAH exposure compared to those in cutotype 1.

**Figure 6:**
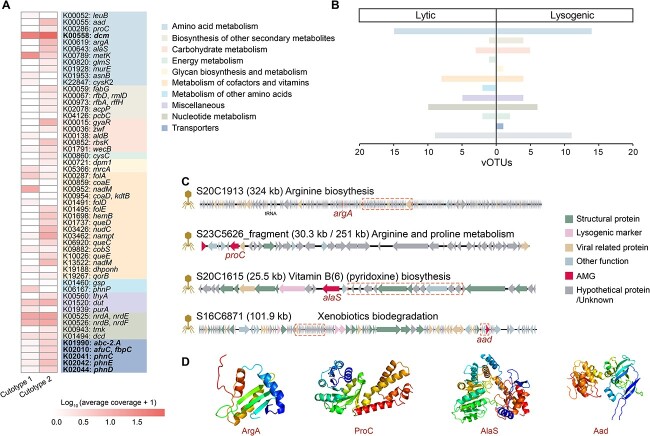
Phage-encoded AMGs in the two cutotypes. (A) Heatmap of the average coverage of each AMG and its Kyoto Encyclopedia of genes and genomes (KEGG) functional annotation. The color indicates the KEGG pathway level. Bold font indicates KEGG orthology (KO) with significant differences between cutotypes (Wilcoxon rank sum test, two-sided, *P*_adj_ < .05). (B) Number of viral operational taxonomic units carrying AMGs with a lysogenic or lytic lifestyle, with the KEGG function indicated. (C) Genomic context of four bacteriophage-encoded AMGs in viruses from cutotype 2 that may aid the bacterial host in adapting to high exposure to polyaromatic hydrocarbons. The AMGs are indicated and the matched fragment (with an identity ≥90%) between a virus and its linked host is indicated by a dashed box. (D) Protein structures of the four bacteriophage-encoded AMGs.

Despite the hosts having CRISPR/Cas systems to protect against viral infection, they appeared to be infected with bacteriophages, possibly due to the presence of small protein inhibitors of CRISPR/Cas systems. Only 2 of the 357 predicted Acr homologs could be matched to a known Acr protein. A similar number of Acr homologs was found in cutotype 1 (*n* = 182) and cutotype 2 (*n* = 175). To explore the phylogeny of the 355 unclassified Acr homologs, a maximum likelihood tree was constructed. This revealed that 15 unclassified homologs clustered closely with known Acr proteins (five with AcrIF and others with diverse subtypes), and the rest were mostly distantly related to AcrIE and AcrIF (Fig. S8 and Table S7). However, the levels of PAH exposure did not affect the phylogenetic distribution of Acr homologs (Fig. S8).

### Phage-encoded AMGs were associated with high-PAH exposure

To determine the potential role of bacteriophages in the metabolic responses of their hosts by cutotype, the diversity and functions of bacteriophage-encoded AMGs were investigated. This identified 153 AMGs encoding 61 types of metabolic functions (Fig. 6A and Table S8), with the most common AMGs in bacteriophages with lysogenic or lytic lifestyles linked to the metabolism of some amino acids (Fig. 6B). The AMGs involved in the biosynthesis of secondary metabolites, carbohydrate metabolism, glycan biosynthesis and metabolism, and transporters were more abundant in bacteriophages with a lysogenic lifestyle than in those with a lytic lifestyle (Fig. 6B). In contrast, AMGs associated with the metabolism of cofactors and vitamins, other amino acids, and nucleotides were more abundant in bacteriophages with a lytic lifestyle than in those with a lysogenic lifestyle (Fig. 6B).

Several AMGs carried by bacteriophages with a higher average coverage in cutotype 2 than cutotype 1, such as *phnCDE* (which encode proteins for phosphonate/phosphate transporters) and *queCDE* (which encode proteins for queuosine biosynthesis), were located together in the same genomic region and shared the same promoter and terminator (Fig. 6A and Fig. S9). Five AMGs associated with transporters were enriched in cutotype 2 over cutotype 1 (WRST, two-sided, *P* = .016; Fig. 6A). An AMG encoding an aryl-alcohol dehydrogenase (K00055 [*aad*]) involved in xenobiotic biodegradation [76] was also identified within a bacteriophage that infected a bacterium *Citricoccus terreus* [LOR019Cbin1] enriched in cutotype 2 (Fig. 6C and Table S8), indicating that the AMG was integrated into the host genome. Similarly, AMGs involved in the biosynthesis of arginine (K00619 [*argA*] encoding an amino-acid N-acetyltransferase) and proline metabolism (K00286 [*proC*] encoding a pyrroline-5-carboxylate reductase) were present in bacteriophages that infected bacteria (*Brevundimonas* sp. [LOR281Cbin1] and *Haematobacter massiliensis* [LOR022Cbin4], respectively) enriched in cutotype 2 (Fig. 6C and Table S8). In addition, other AMGs associated with the metabolism of cofactors and vitamins, such as the biosynthesis of vitamin B (K00643 [*alaS*] encoding a 5-aminolevulinate synthase), were observed in bacteriophages that infected bacteria enriched in cutotype 2 (Fig. 6C and Table S8). The potential functions of all AMGs were further supported by high-confidence protein structure predictions (Table S8). For example, the predicted structure of bacteriophage-encoded ArgA, ProC, AlaS, and Aad showed 99.7% confidence with 34% identity, 100% confidence with 36% identity, 100% confidence with 61% identity, and 100% confidence with 33% identity, respectively, to the reference sequences (Fig. 6C), suggesting that the predicted functions of these AMGs are similar to the references.

## Discussion

Viruses, including bacteriophages, play a crucial role in shaping the diversity and composition of the human skin microbiome both in healthy individuals and those with diseases such as atopic dermatitis [20]. Investigating how cutotype influences skin viromes and virus–host ecological dynamics will help us better understand the interactions within microbiomes in the context of air pollution exposure. In this study, we investigated the relationships between the skin viromes and the two previously categorized cutotypes of the microbiomes in cheek skin samples collected from 124 female Chinese participants with different skin phenotypes under PAH exposure (Fig. 1) [15]. Specifically, participants of cutotype 2 who experienced high PAH exposure exhibited dry skin and hyperpigmentation, which are indicators of an accelerated skin aging process [24], whereas those belonging to cutotype 1, who experienced low PAH exposure, exhibited normal-to-high sebum concentrations and normal pigmentation. The distinct viral diversity and taxonomic and functional compositions between the cutotypes supported the hypothesis that PAH exposure affected the skin viromes. Moreover, we found that virus–host interactions at different levels of PAH exposure were key modulators of bacterial populations, including certain biodegrading bacteria, and may enhance their metabolism via bacteriophage-encoded AMGs.

Viruses affiliated with the class *Caudoviricetes* and the family *Papillomaviridae* were dominant on the cheeks of the participants who were long-term residents of two cities in China. These viruses are also commonly found in different body sites and on the cheeks of individuals from North America [2], South Africa [5], and Australia [20], which suggests that they are important for maintaining skin health and homeostasis. However, there were taxonomic differences in the viruses between cutotypes, such as a high relative abundance of viruses from the family *Zierdtviridae* in cutotype 2 under high PAH exposure. Members of *Zierdtviridae* were also previously found to be enriched in metal-contaminated soils [77], suggesting that these viruses may be selected for polluted environments. There have been no other investigations on the influence of pollutant exposure on the human virome, but a study on the earthworm gut showed a pattern of bacteria–virus diversity under high benzo[*a*]pyrene exposure [22] that contrasts with the pattern observed in this study, i.e. high bacterial diversity and low viral diversity in cutotype 2. This contrast may be attributable to differences in the concentrations and types of pollutant exposure: in this study, under high-exposure conditions, those with cutotype 2 were differentially exposed to 10 different high-molecular-weight PAHs in a total concentration approximately four orders of magnitude lower than that of the single pollutant (benzo[*a*]pyrene) in the earthworm gut [22].

Prey–predator relationships between viruses and their bacterial hosts occur in many ecosystems, such as marine [78], terrestrial [49], and human gut [79] ecosystems, where viral infection can result in different outcomes (i.e. lysis, pseudolysogeny, or resistance) depending on host lineage [80]. Exposure to pollutants upregulates the expression of VFGs in some bacteria in the guts of mice [81], potentially increasing their pathogenicity [82]. In cutotype 2, the enriched rMAGs carried many VFGs, the interactions between viruses and potential bacterial pathogens carrying VFGs were frequent, and the viruses linked to these potential pathogenic hosts tended to have a lytic lifestyle. Viral-mediated lysis of potential pathogens may alter the balance and composition of microbiomes [83], potentially influencing skin health [19]. Moreover, identified viruses could be utilized as an alternative to antibiotics, e.g. in bacteriophage therapy to target skin pathogens, especially antibiotic-resistant bacteria [84]. In addition, we found evidence that a human pathogen (*A. temperans*) in cutotype 2 could use the CRISPR/Cas immune system to defend itself against infection by a specific bacteriophage. Nonetheless, this bacterium remained susceptible to lytic infection by another bacteriophage, indicating the continuous war between bacteriophages and their potential hosts in skin and the role of CRISPR/Cas systems in generating bacteriophage genetic diversity [85]. Furthermore, although the number of CRISPR/Cas systems was higher in cutotype 2 than in cutotype 1, there was no substantial difference in the number of bacteriophage-carried Acr proteins between cutotypes, suggesting that bacteriophages may have also evolved diverse strategies (e.g. antirestriction proteins) to evade or overcome bacterial defense mechanisms [75].

The interactions between viruses and bacteria often result in the modulation of bacterial host abundance, and these relationships can be described by several ecological models [86]. The “Piggyback-the-Winner” (PtW) [87] model explains the increased occurrence of lysogenic infection, which primarily targets fast-growing hosts (denoted “winners”), resulting in high abundances of both a virus and its host. Analogously, the PtW model suggests that increased occurrences of lytic infection primarily target less abundant hosts. In contrast, the “Piggyback-the-Persistent” (PtP) [88] or “Piggyback-the-Loser” (PtL) [89] model explains lysogenic infection that targets less abundant but continuously present hosts (denoted “persisters”) or slow-growing hosts (denoted “losers”). In the present study, the virus–host interactions in cutotypes 1 and 2 could be described by the PtW and PtP or PtL models, respectively, suggesting that environmental stresses and host ecological functions might have driven this differentiation. In this context, *Cutibacterium* emerged as the most abundant and dominant taxon in cutotype 1, positioning it as the “winner.” With a VHR of <1, lysogenic infections were prevalent, allowing viruses to piggyback on *C. acne* strains and other *Cutibacterium* species. *C. acnes* is a key contributor to local skin homeostasis, and its decreased abundance is associated with reduced skin hydration [90] and increased skin disease severity [91]. Although frequent interactions between viruses and *C. acnes* have been observed on normal human skin [2, 80], including samples of cutotype 1, further investigation is needed to understand the specific skin and/or environmental conditions under which viruses are likely to piggyback on linked *C. acnes*. However, many of the low-abundance genera in cutotype 1 had a VHR > 1, suggesting that lytic infection was prevalent and viral lysis played a role in modulating their abundances. In contrast, many of the low-abundance genera in cutotype 2 had a VHR close to 1, substantially lower than that of the low-abundance hosts in cutotype 1, suggesting that lysogenic infection was prevalent in cutotype 2 and these hosts could be considered “persisters” or “losers.” Many of these hosts, such as *Janibacter* spp. [73], *Marmoricola* spp. [74], and *Paracoccus* spp. [92] could biodegrade PAHs. Similarly, in a chlorinated-hydrocarbon-contaminated aquifer, virus–host interactions followed the PtP or PtL model [88]: the less abundant “persisters” exhibiting efficient transformation of contaminants were more prone than “winners” to lysogenic infection by viruses.

Bacteriophages can insert AMGs into their hosts that help them adapt to their environment [22, 33, 93]. In this study, we found evidence that an AMG encoding an aryl-alcohol dehydrogenase [76] was inserted into a bacterium in cutotype 2 under high-PAH exposure, suggesting that this enzyme may help the bacterium to biodegrade xenobiotics. Similarly, an increased presence of AMGs encoding enzymes such as aldehyde dehydrogenase and L-2-haloacid dehalogenase has been observed in organochlorine-contaminated soil samples [93]. These AMGs potentially confer functions related to pesticide degradation on the bacteria, suggesting that the metabolic functions of phage-encoded AMGs are linked to adaptation to specific environments. In contrast, not all phage-encoded AMGs are functional or contribute to their bacterial host metabolism [22]. This was the case with the presence of AMGs (i.e. *argA* and *proC*) for arginine biosynthesis in some bacteriophages in the present study, where arginine is a component of filaggrin-derived natural moisturizing factor (NMF) and helps maintain skin hydration [94]. Specifically, although *argA* and *proC* in bacteriophages were linked to their hosts in cutotype 2, there was no evidence that these genes were inserted into their hosts, and they were not enriched in any viruses. This suggests that viruses in cutotype 2 did not influence the metabolism of their hosts and thus did not alter the dry skin phenotype through the production of NMFs. However, in ecosystems such as the upper sediment of the West Pacific Ocean, filamentous bacteriophages have been shown to assist their host microbes in arginine synthesis under conditions of high pressure and low temperature [95]. Another example involves an AMG encoding a 5-aminolevulinate synthase for heme biosynthesis [96], which is associated with the production of vitamin B6, a compound essential for skin development and maintenance [97]. This AMG was linked to a bacterium in cutotype 2, but there was no evidence of its insertion into this bacterium. However, although the above-described AMGs appeared to not insert into bacteria, their metabolic functions likely serve as a reservoir for transfer between bacteriophages and hosts when suitable conditions emerge [98].

**Figure 7:**
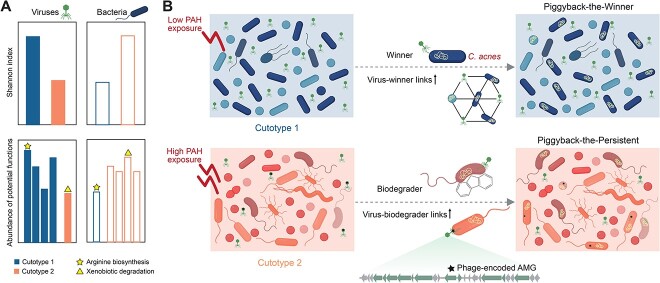
Schematic illustration of the potential mechanistic roles of viruses in cutotypes 1 and 2. (A) Differences in bacterial and viral diversity and functions. (B) Mechanisms of virus–host coevolution.

Taken together, the presence of viruses fulfills varying roles in promoting host skin health when exposed to different levels of PAHs (Fig. 7). Under low levels of PAH exposure in cutotype 1, the skin microbiome exhibited reduced bacterial diversity, with a dominant presence of *C. acnes*, whereas viral diversity was observed to be higher (Fig. 7A). Although bacterial functions in cutotype 1 showed limited enrichment [15], diverse viral functions were observed (Fig. 7A), potentially serving as a genetic reservoir to compensate for the limited bacterial functions. This functional compensation by viruses potentially contributes to the redundancy and resilience of the overall microbiome. Furthermore, the presence of a diverse population of phages with a lysogenic lifestyle, specifically targeting the abundant and dominant *C. acnes* or closely related species (Fig. 7B), suggests their role in regulating the relative abundance of *C. acnes*. This phage-mediated regulation could facilitate the coexistence and equilibrium of *C. acnes*, thereby contributing to the maintenance of normal skin conditions. In contrast, high levels of PAH exposure in cutotype 2 result in skin dryness, likely due to a reduced relative abundance of *C. acnes* and its consequent inability to maintain skin homeostasis. Although the relative abundance of *C. acnes* was reduced, diverse bacteria, albeit mostly in low relative abundances, with a variety of metabolic functions were present [15], and they were targeted by a low diversity of phages (Fig. 7A). These phages carried a low diversity of genes, but among them were genes encoding specific functions, especially those associated with biodegradation (Fig. 7A). During infection, on one hand, phages preferentially infect biodegrading bacteria in a lysogenic manner, thereby supporting the maintenance of these functionally important players (Fig. 7B). In contrast, phages confer specific functions to nonbiodegrading bacteria by inserting AMGs, potentially enhancing their ability to respond to environmental stresses effectively (Fig. 7B).

The present study has shed light on the nature of skin viruses under PAH exposure. Nevertheless, it has a few limitations. First, RNA viruses, which are known to influence skin health, were not considered [99]. Furthermore, owing to the low recovery of fungal sequences in the metagenomes, interactions between fungi and viruses were not considered. Second, the relatively low sequencing depth employed, together with potential biases in the genomic DNA extraction methods, might have led to underestimation of the diversity of viruses and their hosts. Nonetheless, the substantial number of interactions identified between most of the identified viruses and hosts shows that the results are representative. Third, the metagenomic sequencing results did not provide insights into the functionality or defectiveness of the identified viruses and their associated genes, including the role of AMGs in relation to their host bacteria, and the ecological model of each cutotype was interpreted based on *in silico* results. Last, this study did not specifically address mobile genetic elements such as plasmids and bacteriophage-plasmids, which play a pivotal role in the coevolutionary dynamic interactions between bacteriophages and bacteria [100]. Thus, future research should include *in vitro* culturing experiments in a skin model under controlled settings to verify virus–host interactions, determine the specific conditions under which AMGs are inserted and expressed, and identify the bacteriophages that can be used to modulate bacterial abundance under PAH exposure. This may lead to the development of new bacteriophage-based treatments for bacterial infection.

In summary, this study provides the first insights into how the extent of PAH exposure influences the diversity, composition, function, and lifestyle of skin viruses within cutotypes, and into virus–host interactions, which can be described by two different ecological models. The evidence suggests that viruses may mitigate the effects of PAHs on the skin by inserting key functional genes into their bacterial hosts to aid their metabolism. Overall, skin exposure to air pollutants was found to not only alter the skin’s microbiome but also its virome, highlighting the complex interplay between environmental stresses and microbial consortia on the skin. A deeper understanding of this interplay may ultimately lead to the development of strategies to harness the power of viruses to improve skin health under pollution exposure.

## Supplementary Material

Supplementary_Materials_clean_22_Oct_wrae218

TableS1_wrae218

TableS2_wrae218

TableS3_wrae218

TableS4_wrae218

TableS5_wrae218

TableS6_wrae218

TableS7_wrae218

TableS8_wrae218

## Data Availability

The raw paired-end metagenomics sequences and rMAG sequences have been deposited in the National Center of Biotechnology Information’s BioProject under accession number PRJNA730653. The following publicly available databases were used in this study: IMG/VR (https://genome.jgi.doe.gov/portal/IMG_VR/), the Virulence Factor DataBase (http://www.mgc.ac.cn/VFs/), and anti-CRISPRdb (http://guolab.whu.edu.cn/anti-CRISPRdb/). The high-confidence structures and sequences of the AMGs, and the sequences of Acr homologs, are available at https://github.com/Dorothydu12/skin_virome_pah.

## References

[1] Findley K , Oh J, Yang J et al. Topographic diversity of fungal and bacterial communities in human skin. *Nature* 2013;498:367–70. 10.1038/nature12171 23698366 PMC3711185

[2] Saheb Kashaf S , Proctor DM, Deming C et al. Integrating cultivation and metagenomics for a multi-kingdom view of skin microbiome diversity and functions. *Nat Microbiol* 2022;7:169–79. 10.1038/s41564-021-01011-w 34952941 PMC8732310

[3] Hurabielle C , Link VM, Bouladoux N et al. Immunity to commensal skin fungi promotes psoriasiform skin inflammation. *Proc Natl Acad Sci USA* 2020;117:16465–74. 10.1073/pnas.2003022117 32601220 PMC7368261

[4] Hannigan GD , Meisel JS, Tyldsley AS et al. The human skin double-stranded DNA virome: topographical and temporal diversity, genetic enrichment, and dynamic associations with the host microbiome. *MBio* 2015;6:e01578–15. 10.1128/mBio.01578-15 26489866 PMC4620475

[5] van Zyl LJ , Abrahams Y, Stander EA et al. Novel phages of healthy skin metaviromes from South Africa. *Sci Rep* 2018;8:12265. 10.1038/s41598-018-30705-1 30115980 PMC6095929

[6] Belkaid Y , Segre JA. Dialogue between skin microbiota and immunity. *Science* 2014;346:954–9. 10.1126/science.1260144 25414304

[7] Oh J , Byrd AL, Park M et al. Temporal stability of the human skin microbiome. *Cell* 2016;165:854–66. 10.1016/j.cell.2016.04.008 27153496 PMC4860256

[8] Wei Q , Li Z, Gu Z et al. Shotgun metagenomic sequencing reveals skin microbial variability from different facial sites. *Front Microbiol* 2022;13:933189. 10.3389/fmicb.2022.933189 35966676 PMC9364038

[9] Manisalidis I , Stavropoulou E, Stavropoulos A et al. Environmental and health impacts of air pollution: a review. *Front Public Health* 2020;8:505570. 10.3389/fpubh.2020.00014 PMC7044178 32154200

[10] Mallah MA , Changxing L, Mallah MA et al. Polycyclic aromatic hydrocarbon and its effects on human health: an overview. *Chemosphere* 2022;296:133948. 10.1016/j.chemosphere.2022.133948 35151703

[11] Isler MF , Coates SJ, Boos MD. Climate change, the cutaneous microbiome and skin disease: implications for a warming world. *Int J Dermatol* 2023;62:337–45. 10.1111/ijd.16297 35599301

[12] Abolhasani R , Araghi F, Tabary M et al. The impact of air pollution on skin and related disorders: a comprehensive review. *Dermatol Ther* 2021;34:e14840. 10.1111/dth.14840 33527709

[13] Chakravarti D , Venugopal D, Mailander PC et al. The role of polycyclic aromatic hydrocarbon–DNA adducts in inducing mutations in mouse skin. *Mutat Res Genet Toxicol Environ Mutagen* 2008;649:161–78. 10.1016/j.mrgentox.2007.08.007 PMC2254211 17931959

[14] Leung MHY , Tong X, Bastien P et al. Changes of the human skin microbiota upon chronic exposure to polycyclic aromatic hydrocarbon pollutants. *Microbiome* 2020;8:100. 10.1186/s40168-020-00874-1 32591010 PMC7320578

[15] Leung MHY , Tong X, Shen Z et al. Skin microbiome differentiates into distinct cutotypes with unique metabolic functions upon exposure to polycyclic aromatic hydrocarbons. *Microbiome* 2023;11:124. 10.1186/s40168-023-01564-4 37264459 PMC10233911

[16] Sowada J , Schmalenberger A, Ebner I et al. Degradation of benzo[*a*]pyrene by bacterial isolates from human skin. *FEMS Microbiol Ecol* 2014;88:129–39. 10.1111/1574-6941.12276 24372170

[17] Li Z , Xia J, Jiang L et al. Characterization of the human skin resistome and identification of two microbiota cutotypes. *Microbiome* 2021;9:47. 10.1186/s40168-020-00995-7 33597039 PMC7890624

[18] Chen YE , Fischbach MA, Belkaid Y. Skin microbiota–host interactions. *Nature* 2018;553:427–36. 10.1038/nature25177 29364286 PMC6075667

[19] Barnard E , Shi B, Kang D et al. The balance of metagenomic elements shapes the skin microbiome in acne and health. *Sci Rep* 2016;6:39491. 10.1038/srep39491 28000755 PMC5175143

[20] Wielscher M , Pfisterer K, Samardzic D et al. The phageome in normal and inflamed human skin. *Sci Adv* 2023;9:eadg4015. 10.1126/sciadv.adg4015 37774017 PMC10541010

[21] Pamela KC , Christina AK, John HP. Prophage induction of indigenous marine lysogenic bacteria by environmental pollutants. *Mar Ecol Prog Ser* 1998;164:125–33. 10.3354/meps164125

[22] Xia R , Sun M, Balcázar JL et al. Benzo[*a*]pyrene stress impacts adaptive strategies and ecological functions of earthworm intestinal viromes. *ISME J* 2023;17:1004–14. 10.1038/s41396-023-01408-x 37069233 PMC10284932

[23] Palazzi P , Mezzache S, Bourokba N et al. Exposure to polycyclic aromatic hydrocarbons in women living in the Chinese cities of BaoDing and Dalian revealed by hair analysis. *Environ Int* 2018;121:1341–54. 10.1016/j.envint.2018.10.056 30420128

[24] Flament F , Bourokba N, Nouveau S et al. A severe chronic outdoor urban pollution alters some facial aging signs in Chinese women. A tale of two cities. *Int J Cosmet Sci* 2018;40:467–81. 10.1111/ics.12487 30112861

[25] Misra N , Clavaud C, Guinot F et al. Multi-omics analysis to decipher the molecular link between chronic exposure to pollution and human skin dysfunction. *Sci Rep* 2021;11:18302. 10.1038/s41598-021-97572-1 34526566 PMC8443591

[26] Schubert M , Lindgreen S, Orlando L. AdapterRemoval v2: rapid adapter trimming, identification, and read merging. *BMC Research Notes* 2016;9:88. 10.1186/s13104-016-1900-2 26868221 PMC4751634

[27] Edwards JA , Edwards RA. Fastq-pair: efficient synchronization of paired-end fastq files. *bioRxiv* 2019;552885. 10.1101/552885

[28] Wood DE , Lu J, Langmead B. Improved metagenomic analysis with kraken 2. *Genome Biol* 2019;20:257. 31779668 10.1186/s13059-019-1891-0 PMC6883579

[29] Lu J , Breitwieser FP, Thielen P et al. Bracken: estimating species abundance in metagenomics data. *PeerJ Comput Sci* 2017;3:e104. 10.7717/peerj-cs.104 PMC12016282 40271438

[30] Uritskiy GV , DiRuggiero J, Taylor J. MetaWRAP—a flexible pipeline for genome-resolved metagenomic data analysis. *Microbiome* 2018;6:158. 10.1186/s40168-018-0541-1 30219103 PMC6138922

[31] Guo J , Bolduc B, Zayed AA et al. VirSorter2: a multi-classifier, expert-guided approach to detect diverse DNA and RNA viruses. *Microbiome* 2021;9:37. 10.1186/s40168-020-00990-y 33522966 PMC7852108

[32] Ren J , Song K, Deng C et al. Identifying viruses from metagenomic data using deep learning. *Quant Biol* 2020;8:64–77. 10.1007/s40484-019-0187-4 34084563 PMC8172088

[33] Du S , Tong X, Lai ACK et al. Highly host-linked viromes in the built environment possess habitat-dependent diversity and functions for potential virus-host coevolution. *Nat Commun* 2023;14:2676. 10.1038/s41467-023-38400-0 37160974 PMC10169181

[34] Nissen JN , Johansen J, Allesøe RL et al. Improved metagenome binning and assembly using deep variational autoencoders. *Nat Biotechnol* 2021;39:555–60. 10.1038/s41587-020-00777-4 33398153

[35] Johansen J , Plichta DR, Nissen JN et al. Genome binning of viral entities from bulk metagenomics data. *Nat Commun* 2022;13:965. 10.1038/s41467-022-28581-5 35181661 PMC8857322

[36] Nayfach S , Camargo AP, Schulz F et al. CheckV assesses the quality and completeness of metagenome-assembled viral genomes. *Nat Biotechnol* 2021;39:578–85. 10.1038/s41587-020-00774-7 33349699 PMC8116208

[37] Nayfach S , Páez-Espino D, Call L et al. Metagenomic compendium of 189,680 DNA viruses from the human gut microbiome. *Nat Microbiol* 2021;6:960–70. 10.1038/s41564-021-00928-6 34168315 PMC8241571

[38] Hockenberry AJ , Wilke CO. BACPHLIP: predicting bacteriophage lifestyle from conserved protein domains. *PeerJ* 2021;9:e11396. 10.7717/peerj.11396 33996289 PMC8106911

[39] Kieft K , Zhou Z, Anantharaman K. VIBRANT: automated recovery, annotation and curation of microbial viruses, and evaluation of viral community function from genomic sequences. *Microbiome* 2020;8:90. 10.1186/s40168-020-00867-0 32522236 PMC7288430

[40] Tong X , Leung MHY, Shen Z et al. Metagenomic insights into the microbial communities of inert and oligotrophic outdoor pier surfaces of a coastal city. *Microbiome* 2021;9:213. 10.1186/s40168-021-01166-y 34724986 PMC8562002

[41] Olm MR , Brown CT, Brooks B et al. dRep: a tool for fast and accurate genomic comparisons that enables improved genome recovery from metagenomes through de-replication. *ISME J* 2017;11:2864–8. 10.1038/ismej.2017.126 28742071 PMC5702732

[42] Parks DH , Imelfort M, Skennerton CT et al. CheckM: assessing the quality of microbial genomes recovered from isolates, single cells, and metagenomes. *Genome Res* 2015;25:1043–55. 10.1101/gr.186072.114 25977477 PMC4484387

[43] Bowers RM , Kyrpides NC, Stepanauskas R et al. Minimum information about a single amplified genome (MISAG) and a metagenome-assembled genome (MIMAG) of bacteria and archaea. *Nat Biotechnol* 2017;35:725–31. 10.1038/nbt.3893 28787424 PMC6436528

[44] Chaumeil P-A , Mussig AJ, Hugenholtz P et al. GTDB-Tk: a toolkit to classify genomes with the genome taxonomy database. *Bioinformatics* 2020;36:1925–7. 10.1093/bioinformatics/btz848 PMC7703759 31730192

[45] Asnicar F , Thomas AM, Beghini F et al. Precise phylogenetic analysis of microbial isolates and genomes from metagenomes using PhyloPhlAn 3.0. *Nat Commun* 2020;11:2500. 10.1038/s41467-020-16366-7 32427907 PMC7237447

[46] Enright AJ , Van Dongen S, Ouzounis CA. An efficient algorithm for large-scale detection of protein families. *Nucleic Acids Res* 2002;30:1575–84. 10.1093/nar/30.7.1575 11917018 PMC101833

[47] Hyatt D , Chen G-L, LoCascio PF et al. Prodigal: prokaryotic gene recognition and translation initiation site identification. *BMC Bioinformatics* 2010;11:119. 10.1186/1471-2105-11-119 20211023 PMC2848648

[48] Camargo AP , Nayfach S, Chen IMA et al. IMG/VR v4: an expanded database of uncultivated virus genomes within a framework of extensive functional, taxonomic, and ecological metadata. *Nucleic Acids Res* 2023;51:D733–43. 10.1093/nar/gkac1037 36399502 PMC9825611

[49] Emerson JB , Roux S, Brum JR et al. Host-linked soil viral ecology along a permafrost thaw gradient. *Nat Microbiol* 2018;3:870–80. 10.1038/s41564-018-0190-y 30013236 PMC6786970

[50] Aramaki T , Blanc-Mathieu R, Endo H et al. KofamKOALA: KEGG Ortholog assignment based on profile HMM and adaptive score threshold. *Bioinformatics* 2019;36:2251–2. 10.1093/bioinformatics/btz859 PMC7141845 31742321

[51] Shaffer M , Borton MA, McGivern BB et al. DRAM for distilling microbial metabolism to automate the curation of microbiome function. *Nucleic Acids Res* 2020;48:8883–900. 10.1093/nar/gkaa621 32766782 PMC7498326

[52] Burley SK , Berman HM, Kleywegt GJ et al. Protein data Bank (PDB): the single global macromolecular structure archive. *Protein Crystallogr Meth Protoc* 2017;1607:627–41. 10.1007/978-1-4939-7000-1_26 PMC5823500 28573592

[53] Varadi M , Anyango S, Deshpande M et al. AlphaFold protein structure database: massively expanding the structural coverage of protein-sequence space with high-accuracy models. *Nucleic Acids Res* 2022;50:D439–44. 10.1093/nar/gkab1061 34791371 PMC8728224

[54] Kelley LA , Mezulis S, Yates CM et al. The Phyre2 web portal for protein modeling, prediction and analysis. *Nat Protoc* 2015;10:845–58. 10.1038/nprot.2015.053 25950237 PMC5298202

[55] Seemann T . Prokka: rapid prokaryotic genome annotation. *Bioinformatics* 2014;30:2068–9. 10.1093/bioinformatics/btu153 24642063

[56] Huerta-Cepas J , Szklarczyk D, Heller D et al. eggNOG 5.0: a hierarchical, functionally and phylogenetically annotated orthology resource based on 5090 organisms and 2502 viruses. *Nucleic Acids Res* 2018;47:D309–14. 10.1093/nar/gky1085 PMC6324079 30418610

[57] Liu B , Zheng D, Zhou S et al. VFDB 2022: a general classification scheme for bacterial virulence factors. *Nucleic Acids Res* 2022;50:D912–7. 10.1093/nar/gkab1107 34850947 PMC8728188

[58] Chen T , Liu T, Wu Z et al. Virus–pathogen interactions improve water quality along the middle route of the south-to-north water Diversion Canal. *ISME J* 2023;17:1719–32. 10.1038/s41396-023-01481-2 37524909 PMC10504254

[59] Bland C , Ramsey TL, Sabree F et al. CRISPR recognition tool (CRT): a tool for automatic detection of clustered regularly interspaced palindromic repeats. *BMC Bioinformatics* 2007;8:209. 10.1186/1471-2105-8-209 17577412 PMC1924867

[60] Edgar RC . PILER-CR: fast and accurate identification of CRISPR repeats. *BMC Bioinformatics* 2007;8:18. 10.1186/1471-2105-8-18 17239253 PMC1790904

[61] Galiez C , Siebert M, Enault F et al. WIsH: who is the host? Predicting prokaryotic hosts from metagenomic phage contigs. *Bioinformatics* 2017;33:3113–4. 10.1093/bioinformatics/btx383 28957499 PMC5870724

[62] Shannon P , Markiel A, Ozier O et al. Cytoscape: a software environment for integrated models of biomolecular interaction networks. *Genome Res* 2003;13:2498–504. 10.1101/gr.1239303 14597658 PMC403769

[63] Russel J , Pinilla-Redondo R, Mayo-Muñoz D et al. CRISPRCasTyper: automated identification, annotation, and classification of CRISPR-Cas loci. *CRISPR J* 2020;3:462–9. 10.1089/crispr.2020.0059 33275853

[64] Yang B , Zheng J, Yin Y. AcaFinder: genome Mining for Anti-CRISPR-associated genes. *mSystems* 2022;7:e0081722–00822. 10.1128/msystems.00817-22 36413017 PMC9765179

[65] Dong C , Wang X, Ma C et al. Anti-CRISPRdb v2.2: an online repository of anti-CRISPR proteins including information on inhibitory mechanisms, activities and neighbors of curated anti-CRISPR proteins. *Database* 2022;2022:baac010. 10.1093/database/baac010 35348649 PMC9248852

[66] Deorowicz S , Debudaj-Grabysz A, Gudyś A. FAMSA: fast and accurate multiple sequence alignment of huge protein families. *Sci Rep* 2016;6:1–13. 10.1038/srep33964 27670777 PMC5037421

[67] Capella-Gutiérrez S , Silla-Martínez JM, Gabaldón T. trimAl: a tool for automated alignment trimming in large-scale phylogenetic analyses. *Bioinformatics* 2009;25:1972–3. 10.1093/bioinformatics/btp348 19505945 PMC2712344

[68] Duca R-C , Hardy E, Salquèbre G et al. Hair decontamination procedure prior to multi-class pesticide analysis. *Drug Test Anal* 2014;6:55–66. 10.1002/dta.1649 24817049

[69] Dixon P . VEGAN, a package of R functions for community ecology. *J Veg Sci* 2003;14:927–30. 10.1111/j.1654-1103.2003.tb02228.x

[70] Mallick H , Rahnavard A, McIver LJ et al. Multivariable association discovery in population-scale meta-omics studies. *PLoS Comput Biol* 2021;17:e1009442. 10.1371/journal.pcbi.1009442 34784344 PMC8714082

[71] Kim SY , Lee J, Lee SY. Metabolic engineering of *Corynebacterium glutamicum* for the production of L-ornithine. *Biotechnol Bioeng* 2015;112:416–21. 10.1002/bit.25440 25163446

[72] Lü H , Wei J-L, Tang G-X et al. Microbial consortium degrading of organic pollutants: source, degradation efficiency, pathway, mechanism and application. *J Clean Prod* 2024;451:141913. 10.1016/j.jclepro.2024.141913

[73] Tikilili PV , Nkhalambayausi-Chirwa EM. Characterization and biodegradation of polycyclic aromatic hydrocarbons in radioactive wastewater. *J Hazard Mater* 2011;192:1589–96. 10.1016/j.jhazmat.2011.06.079 21782341

[74] Li J , Peng K, Zhang D et al. Autochthonous bioaugmentation with non-direct degraders: a new strategy to enhance wastewater bioremediation performance. *Environ Int* 2020;136:105473. 10.1016/j.envint.2020.105473 31999970

[75] Hobbs SJ , Wein T, Lu A et al. Phage anti-CBASS and anti-Pycsar nucleases subvert bacterial immunity. *Nature* 2022;605:522–6. 10.1038/s41586-022-04716-y 35395152 PMC9117128

[76] Lopez ES , Elufisan TO, Bustos P et al. Complete genome report of a hydrocarbon-degrading *Sphingobium yanoikuyae* S72. *Appl Sci* 2022;12:6201. 10.3390/app12126201

[77] Zhang H , Huang J, Zeng W et al. Dissecting the metal resistance genes contributed by virome from mining-affected metal contaminated soils. *Front Environ Sci* 2023;11:1182673. 10.3389/fenvs.2023.1182673

[78] Coutinho FH , Silveira CB, Gregoracci GB et al. Marine viruses discovered via metagenomics shed light on viral strategies throughout the oceans. *Nat Commun* 2017;8:15955. 10.1038/ncomms15955 28677677 PMC5504273

[79] Liang G , Zhao C, Zhang H et al. The stepwise assembly of the neonatal virome is modulated by breastfeeding. *Nature* 2020;581:470–4. 10.1038/s41586-020-2192-1 32461640 PMC7263352

[80] Liu J , Yan R, Zhong Q et al. The diversity and host interactions of *Propionibacterium acnes* bacteriophages on human skin. *ISME J* 2015;9:2078–93. 10.1038/ismej.2015.47 25848871 PMC4542041

[81] Viennois E , Bretin A, Dubé PE et al. Dietary emulsifiers directly impact adherent-invasive *E. coli* gene expression to drive chronic intestinal inflammation. *Cell Rep* 2020;33:108229. 10.1016/j.celrep.2020.108229 33027647 PMC7539532

[82] Haj Ishak Al Ali R , Mondamert L, Berjeaud J-M et al. Application of QSAR approach to assess the effects of organic pollutants on bacterial virulence factors. *Microorganisms* 2023;11:1375. 10.3390/microorganisms11061375 37374877 PMC10301662

[83] Chatterjee A , Duerkop BA. Beyond bacteria: bacteriophage-eukaryotic host interactions reveal emerging paradigms of health and disease. *Front Microbiol* 2018;9:381438. 10.3389/fmicb.2018.01394 PMC6030379 29997604

[84] Kortright KE , Chan BK, Koff JL et al. Phage therapy: a renewed approach to combat antibiotic-resistant bacteria. *Cell Host Microbe* 2019;25:219–32. 10.1016/j.chom.2019.01.014 30763536

[85] Hampton HG , Watson BNJ, Fineran PC. The arms race between bacteria and their phage foes. *Nature* 2020;577:327–36. 10.1038/s41586-019-1894-8 31942051

[86] Brown TL , Charity OJ, Adriaenssens EM. Ecological and functional roles of bacteriophages in contrasting environments: marine, terrestrial and human gut. *Curr Opin Microbiol* 2022;70:102229. 10.1016/j.mib.2022.102229 36347213

[87] Silveira CB , Rohwer FL. Piggyback-the-winner in host-associated microbial communities. *NPJ Biofilms Microbiomes* 2016;2:16010. 10.1038/npjbiofilms.2016.10 28721247 PMC5515262

[88] Paterson JS , Smith RJ, McKerral JC et al. A hydrocarbon-contaminated aquifer reveals a piggyback-the-persistent viral strategy. *FEMS Microbiol Ecol* 2019;95:fiz116. 10.1093/femsec/fiz116 31314089

[89] Knowles B , Silveira CB, Bailey BA et al. Lytic to temperate switching of viral communities. *Nature* 2016;531:466–70. 10.1038/nature17193 26982729

[90] Almoughrabie S , Cau L, Cavagnero K et al. Commensal *Cutibacterium acnes* induce epidermal lipid synthesis important for skin barrier function. *Sci Adv* 2023;9:eadg6262. 10.1126/sciadv.adg6262 37595033 PMC10438445

[91] Rozas M , Hart de Ruijter A, Fabrega MJ et al. From dysbiosis to healthy skin: major contributions of *Cutibacterium acnes* to skin homeostasis. *Microorganisms* 2021;9:628. 10.3390/microorganisms9030628 33803499 PMC8003110

[92] Phale PS , Malhotra H, Shah BA. Degradation strategies and associated regulatory mechanisms/features for aromatic compound metabolism in bacteria. In: Gadd G.M., Sariaslani S. (eds.), Advances in Applied Microbiology. Cambridge: Academic Press, 2020, 1–65 10.1016/bs.aambs.2020.02.002. 32762865

[93] Zheng X , Jahn MT, Sun M et al. Organochlorine contamination enriches virus-encoded metabolism and pesticide degradation associated auxiliary genes in soil microbiomes. *ISME J* 2022;16:1397–408. 10.1038/s41396-022-01188-w 35039616 PMC9038774

[94] Kubo A , Ishizaki I, Kubo A et al. The stratum corneum comprises three layers with distinct metal-ion barrier properties. *Sci Rep* 2013;3:1731. 10.1038/srep01731 23615774 PMC3635058

[95] Jian H , Xiong L, Xu G et al. Filamentous phage SW1 is active and influences the transcriptome of the host at high-pressure and low-temperature. *Environ Microbiol Rep* 2016;8:358–62. 10.1111/1758-2229.12388 26929122

[96] Heinemann IU , Jahn M, Jahn D. The biochemistry of heme biosynthesis. *Arch Biochem Biophys* 2008;474:238–51. 10.1016/j.abb.2008.02.015 18314007

[97] Kato N . Role of vitamin B6 in skin health and diseases. In: Preedy V.R. (ed.), Handbook of Diet, Nutrition and the Skin. Wageningen: Wageningen Academic Publishers, 2012, 58–66. 10.3920/9789086867295_005.

[98] Tan Y , Yu P, Huang D et al. Enhanced bacterium–phage symbiosis in attached microbial aggregates on a membrane surface facing elevated hydraulic stress. *Environ Sci Technol* 2023;57:17324–37. 10.1021/acs.est.3c05452 37930060

[99] Kawamura T , Ogawa Y, Aoki R et al. Innate and intrinsic antiviral immunity in skin. *J Dermatol Sci* 2014;75:159–66. 10.1016/j.jdermsci.2014.05.004 24928148

[100] Pfeifer E , Rocha EPC. Phage-plasmids promote recombination and emergence of phages and plasmids. *Nat Commun* 2024;15:1545. 10.1038/s41467-024-45757-3 38378896 PMC10879196

