# Supplementary material for: Chronic exposure to polycyclic aromatic hydrocarbons alters skin virome composition and virus–host interactions
Source: ISME J. 2024 Oct 25;18(1):wrae218. doi: 10.1093/ismejo/wrae218 (PMC11549919; doi:10.1093/ismejo/wrae218)
Supplement: Supplementary_Materials_clean_22_Oct_wrae218 [file supplementary_materials_clean_22_oct_wrae218.docx]

**Supplementary Information for**

**Chronic exposure to polycyclic aromatic hydrocarbons alters skin virome composition and virus–host interactions**

Shicong Du,^1^ Xinzhao Tong,^1,2^ Marcus H. Y. Leung,^1^ Richard J. Betts,^3^ Anthony C. Woo,^4^ Philippe Bastien,^4^ Namita Misra,^4^ Luc Aguilar,^4^ Cécile Clavaud,^4^ and Patrick K. H. Lee^5,6^*

^1^School of Energy and Environment, City University of Hong Kong, Kowloon, Hong Kong SAR, China

^2^Department of Biological Sciences, School of Science, Xi'an Jiaotong-Liverpool University, Suzhou, P. R. China

^3^L’Oréal Research and Innovation, Singapore

^4^L’Oréal Research and Innovation, Aulnay-Sous-Bois, France

^5^School of Energy and Environment and State Key Laboratory of Marine Pollution, City University of Hong Kong, Hong Kong SAR, China

^6^Low-Carbon and Climate Impact Research Centre, City University of Hong Kong, Hong Kong SAR, China

Correspondence: *B5423, Yeung Kin Man Academic Building, School of Energy and Environment, City University of Hong Kong, Tat Chee Avenue, Kowloon, Hong Kong SAR, China; E-mail: patrick.kh.lee@cityu.edu.hk; Tel: (852) 3442-4625; Fax: (852) 3442-0688.

Supplemental information includes:

**Supplementary Figure S1 to S9**

**Table legend of Table S1 to S8**

**Fig. S1. Overview of the skin viromes.** (**A**) Comparison of the numbers and genome lengths of complete, medium- and high-quality viruses identified by Virsorter2 (Vs2), DeepVirFinder (DVF), and PHAMB. The different colors indicate shared viruses identified by both Vs2 and DVF (red), unique viruses identified by DVF (green) or Vs2 (purple), and viruses identified by PHAMB (blue). The combination of Vs2 and DVF identified 30 complete, 57 medium- and 49 high-quality viruses, whereas PHAMB identified 19 complete, 125 medium- and 120 high-quality viruses. (**B**) Accumulation curves of the viral bins in the 124 cheek skin samples. The confidence intervals derived from the standard deviations are shown in light blue. (**C**) Accumulation curves of the viral bins clustered at the species, genus, and family levels. In each box-and-whisker plot, the box indicates the median, the first quartile, and the third quartile; the whiskers span 1.5 times the interquartile range; and the diamond indicates the mean. Points that lie beyond the whiskers indicate outliers.

**Fig. S2. Measured concentrations of polycyclic aromatic hydrocarbons (PAHs) in hair samples from the participants.** (**A**) The concentrations (log_10_ scale) of high- and low-molecular-weight PAHs are shown in purple text and green text, respectively, for cutotypes 1 and 2. A molecular weight cut-off of 200 g/mol was used to distinguish the two groups of PAHs. (**B**) Total average concentrations (log_10_ scale) of high- and low-molecular-weight PAHs in cutotypes 1 and 2. Only those with *p*_adj_ < 0.05 (Wilcoxon rank sum test, two-sided) are indicated on the figure. In each box-and-whisker plot, the box indicates the median, the first quartile, and the third quartile; the whiskers span 1.5 times the interquartile range; and the diamond indicates the mean. Points that lie beyond the whiskers are outliers.

**Fig. S3. Diversity, taxonomy and lifestyle of viral operational taxonomic units (vOTUs).** (**A**) Lifestyles of viruses in the two cutotypes (i.e., lysogenic (*n* = 62) or lytic (*n* = 110)). (**B**) Alpha-diversity indexes (i.e., Shannon’s *H* and Pielou’s *J* evenness) of the vOTUs between cutotypes 1 and 2. (**C**) Enrichment of vOTUs (*n* = 37) between cutotypes according to MaAsLin2. The viral lifestyle and taxonomy of each vOTU are indicated. (**D**) Heatmap of the average coverage (log_10_ scale) of the vOTUs organized by cutotype across the samples. The pie chart shows the proportion of genera within the unclassified family of *Caudoviricetes*. In the label text, the prefixes “c” and “f” indicate the lowest possible assigned taxonomic ranks at the class and family levels, respectively. In each box-and-whisker plot, the box indicates the median, the first quartile, and the third quartile; the whiskers each span 1.5 times the interquartile range; and the diamond indicates the mean. Points beyond the whiskers indicate outliers.

**Fig. S4. Functional compositions of viral operational taxonomic units (vOTUs).** (**A**) Procrustes analysis showing the congruence between the taxonomic and functional compositions of the vOTUs. (**B**) Accumulation curve of the open reading frames (ORFs) of vOTUs. (**C**) Functional annotation of the ORF clusters based on the Kyoto Encyclopedia of Genes and Genomes (KEGG) (only clusters with > 10 genes are shown).

**Fig. S5.** **Representative metagenome-assembled genomes (rMAGs) reconstructed from 124 cheek skin samples.** (**A**) Phylogenetic tree depicting 112 rMAGs. In the labels, the prefix “s” indicates a known species, while the prefixes “g” and “f” indicate the lowest possible assigned taxonomic ranks at the genus and family levels, respectively. rMAGs that could not be assigned to a known species are marked with a red star in the inner ring. Enrichment of rMAGs between cutotypes, based on MaAsLin2 analysis, is shown in the second outer ring. The type of clustered regularly interspaced short palindromic repeat-associated protein identified in each rMAG is indicated in the outermost ring. (**B**) Number of virulence factor genes identified in the rMAGs at the genus level. (**C**) Network illustrating viruses and their predicted bacterial hosts at the genus level. Circles and diamonds represent predicted bacterial and viral hosts, respectively, and the edges of shapes are color-coded based on the prediction method used. (**D**) The frequency of virus–host links in the samples at the genus level of hosts.

**Fig. S6** The average relative abundance of viruses carrying lysogenic (integrase) or lytic ((endo)lysin, holin, and/or peptidoglycan hydrolase) markers linked to two potential biodegrader genera, *Janibacter* and *Marmoricola*.

**Fig. S7. Relationships between the concentrations of polyaromatic hydrocarbons (PAHs) and virus-to-host abundance ratios (VHRs).** (**A**) Heatmap showing Pearson’s correlations between the concentrations of PAHs and selected representative metagenome-assembled genomes (rMAGs) (i.e., genera whose VHRs differed significantly between cutotypes). High- and low-molecular-weight PAHs are represented by purple- and green-colored text, respectively. The rMAGs enriched in cutotype 2 are colored dark blue. An asterisk (*) denotes *p* < 0.05, based on the corresponding Pearson’s coefficient. (B) Largest Pearson’s correlation between the average VHR of the rMAGs shown in panel (**A**) and the measured concentrations of PAHs (excluding nicotine and cotinine). The results of LOR295Cbin.5 and LOR282Cbin.5 are shown in **Fig. 4E**.

**Fig. S8. Maximum-likelihood phylogenetic tree of the 357 Acr homologs identified in this study, along with the 3,690 Acr proteins obtained from a curated anti-CRISPR database.** The red circle represents the predicted Acr proteins that are most distantly related to known Acr proteins. The branch colors indicate the sample origins, with cutotype 1 in blue and cutotype 2 in orange.

**Fig. S9. Genomic context and protein structure of the seven auxiliary metabolic genes carried by two bacteriophages in cutotype 2.** An arrow and a hollow circle indicate the promoter and terminator, respectively. The structures are color-coded from the N- to the C-terminus.

Table S1. (separate file)

Table S1. Information on the putative and high-quality viral bins.

Table S2. (separate file)

Table S2. Canonical correlation analysis of the relationships between the concentrations of polyaromatic hydrocarbons (PAHs) and the viral operational taxonomic units (vOTUs) detected in cheek skin samples.

Table S3. (separate file)

Table S3. Factors that explain taxonomic and functional differences between viral operational taxonomic units (vOTUs) detected in cheek skin samples.

Table S4. (separate file)

Table S4. Enrichment of viral functions between cutotypes according to MaAsLin2 and the correlations between specific enriched KOs and their corresponding metabolites.

Table S5. (separate file)

Table S5. Information on the 112 representative metagenome-assembled genomes (rMAGs).

Table S6. (separate file)

Table S6. Predicted hosts for viruses and Pearson’s correlations between viral operational taxonomic units (vOTUs) and representative metagenome-assembled genomes (rMAGs).

Table S7. (separate file)

Table S7. Properties of the predicted anti-clustered regularly interspaced short palindromic repeats (CRISPR) (Acr) proteins, anti-CRISPR-associated (Aca) proteins, and Aca-like proteins in the viral operational taxonomic units detected in cheek skin samples.

Table S8. (separate file)

Table S8. Properties of the phage-encoded auxiliary metabolic genes (AMGs) in the skin viral operational taxonomic units (vOTUs).
